# Association between Baseline Subfoveal Choroidal Thickness and Anatomical and Functional Outcomes in Geographic Atrophy

**DOI:** 10.1016/j.xops.2025.100986

**Published:** 2025-10-27

**Authors:** Alythia Vo, Liangbo Linus Shen, Irene Pak, Abu Tahir Taha, Antonio Z. Diaz, Jay M. Stewart

**Affiliations:** 1 Department of Ophthalmology, University of California, San Francisco, San Francisco, California; 2 Department of Ophthalmology, Zuckerberg San Francisco General Hospital and Trauma Center, San Francisco, California; 3 Department of Ophthalmology, Duke University School of Medicine, Durham, North Carolina

**Keywords:** Age-related macular degeneration, Geographic atrophy, Subfoveal choroidal thickness

## Abstract

**Objective:**

To investigate the relationship between baseline subfoveal choroidal thickness (SFChT) and both visual outcomes and geographic atrophy (GA) growth rate, and to assess whether SFChT mediates the treatment effect of oral metformin on GA progression.

**Design:**

Secondary analysis of a randomized controlled trial.

**Participants:**

Seventy eyes (34 metformin; 36 observation) from 44 participants (21 metformin; 23 observation) with GA and ≥6 months of follow-up in the METformin for the MINimization of Geographic Atrophy Progression study.

**Methods:**

Subfoveal choroidal thickness was measured from baseline OCT. We calculated GA area growth rate by subtracting the GA area at the first visit from the GA area at the last visit and dividing the result by the time interval. Geographic atrophy perimeter-adjusted growth rate was calculated by dividing GA area growth rate by the mean GA perimeter between the first and last visit.

**Main Outcome Measures:**

Longitudinal changes in GA area and visual acuity.

**Results:**

Baseline SFChT was not significantly associated with baseline GA area (*P* = 0.51), baseline best-corrected visual acuity (BCVA) (*P* = 0.49), baseline low-luminance visual acuity (LLVA) (*P* = 0.85), or rim area focal hyperautofluorescence signals (*P* = 0.29). Baseline SFChT was not significantly associated with GA perimeter-adjusted growth rate (*P* = 0.74), the decline rate of BCVA (*P* = 0.14), and the decline rate of LLVA (*P* = 0.71). However, sensitivity analyses in GA subgroups found that baseline SFChT was associated with decreased rate of BCVA decline in patients with foveal-involving GA (Spearman ρ = 0.03, *P* = 0.03). Baseline SFChT did not significantly influence the effect of oral metformin on GA perimeter-adjusted growth rate (*P* = 0.78).

**Conclusions:**

Greater baseline SFChT was significantly associated with slower BCVA decline in eyes with foveal-involving GA, suggesting a possible localized role of choroidal thickness in preserving central vision. However, SFChT was not associated with GA growth rate, LLVA decline, or baseline anatomical and functional measures. It also did not mediate the effect of oral metformin. While SFChT lacks prognostic value for GA progression overall, it may hold limited relevance for central vision outcomes in foveal-involving GA.

**Financial Disclosure(s):**

Proprietary or commercial disclosure may be found in the Footnotes and Disclosures at the end of this article.

Geographic atrophy (GA) is an advanced form of nonexudative age-related macular degeneration (AMD) that affects approximately 5 million individuals worldwide.^1^ Geographic atrophy leads to gradual vision loss due to the progressive deterioration of photoreceptors, retinal pigment epithelium, Bruch membrane, and the choriocapillaris (CC).^2^^,^^3^ In 2023, the United States Food and Drug Administration approved the use of pegcetacoplan and avacincaptad pegol for the treatment of GA secondary to AMD.^4^, ^5^, ^6^ Clinical trials evaluating other therapies are also ongoing.^7^

While the exact pathophysiological mechanism for GA progression remains unclear, multiple studies have identified various imaging biomarkers associated with GA onset and progression.^8^ Of these, subfoveal choroidal thickness (SFChT) is a potential OCT biomarker of GA severity and progression rate because SFChT may reflect choroidal perfusion status and vascular integrity.^9^, ^10^, ^11^, ^12^, ^13^ Choroidal changes in various stages of AMD have been identified by several studies. In early stage AMD, subtle changes in CC attenuation and narrowing have been described, with progressive capillary decline and CC loss in more advanced stages.^14^, ^15^, ^16^, ^17^ Choriocapillaris density has also been found to be inversely correlated with the extent of drusen and pseudodrusen, suggesting that vascular endothelial cell loss occurs in association with drusen formation.^18^ Prior work using laser Doppler flowmetry has also noted a progressive decline in choroidal volume, flow, and velocity with increasing disease severity.^19^ Thinning of the choroid, as indicated by reduced SFChT, may reflect underlying choroidal vascular insufficiency, which can exacerbate retinal degeneration and promote the expansion of GA. In early stage AMD, SFChT may show little or no change.^20^ However, the relationship between SFChT and late stage AMD with GA remains controversial in the literature. Some studies have reported a significant negative correlation between baseline GA area or best-corrected visual acuity (BCVA) and SFChT,^12^^,^^21^, ^22^, ^23^ while others have found no significant association.^24^, ^25^, ^26^

Prior studies have also investigated hyperautofluorescent signals in the junctional zone of GA lesions as a potential biomarker in AMD, with some suggesting that hyperautofluorescence patterns may be associated with GA progression.^27^, ^28^, ^29^, ^30^ Lindner et al^26^ stratified GA subtypes based on perilesional fundus autofluorescence (FAF) patterns and reported a significantly thinner choroid in the “diffuse-trickling” GA subtype. However, grading these patterns is relatively subjective and may lead to varied reproducibility, prompting a shift toward quantitative measures of hyperautofluorescence.^31^, ^32^, ^33^, ^34^ Bearelly et al defined rim area focal hyperautofluorescence (RAFH) as the percentage of area with increased autofluorescence within the 500-μm border around GA.^31^ More recently, Allingham et al^32^ developed and validated a software that allows semiautomatic quantification of RAFH, defined as a ratio of hyperautofluorescent areas over the total amount of area enclosed within a 450 μm border around GA. Higher baseline RAFH was associated with faster GA progression; however, its association with SFChT has not yet been explored.^32^^,^^34^

The association between SFChT and GA progression has also been explored in prior studies, though findings remain inconsistent. Some studies have reported a faster GA progression in eyes with thinner SFChT at baseline,^10^^,^^12^ while others have found no significant relationship.^13^^,^^35^ It is plausible that the discrepancy may be confounded by baseline morphological factors such as lesion size, number of lesions, and circularity index.^29^, ^33^^,^^36^, ^37^, ^38^, ^39^, ^40^, ^41^, ^42^, ^43^ To address these confounding factors, our prior work has demonstrated that GA perimeter-adjusted growth rate provides a more robust assessment by accounting for baseline GA area, number of lesions, and circularity index.^44^ However, prior studies have not specifically examined the relationship between SFChT and perimeter-adjusted GA growth rate.

Our group previously conducted the METformin for the MINimization of Geographic Atrophy Progression (METforMIN) trial, which found that oral metformin did not significantly slow GA progression.^45^ However, it remains unclear whether patients with a thicker choroid may have increased drug delivery due to enhanced blood flow, potentially mediating the treatment effect. Additionally, given the inconsistent findings regarding SFChT as a biomarker for GA progression, we performed a secondary analysis of the METforMIN trial to investigate the relationship between SFChT and multiple anatomical and functional outcomes in GA, as well as to determine whether SFChT mediates the treatment effect of oral metformin.

## Methods

### Study Design and Eligibility Criteria

This study is a secondary analysis of the METforMIN clinical trial (ClinicalTrials.gov identifier: NCT02684578).^45^ This trial was a multicenter phase II clinical trial that investigated the efficacy of oral metformin in slowing down the rate of progression of GA. We recruited nondiabetic participants >55 years of age with GA secondary to nonexudative AMD from 12 clinical sites. Sixty-six eligible patients were randomized in a 1:1 ratio to either metformin or observation. Patients in the metformin arm were instructed to gradually increase the metformin dose to 1000 mg twice daily, which they took for 18 months. Patients in the observation arm were followed every 6 months for 18 months. We evaluated all patients at baseline and every 6 months with a complete ophthalmic examination. Each examination included BCVA using ETDRS chart, low-luminance visual acuity (LLVA) using a 2.0 log unit neutral density filter, FAF imaging, and OCT. The study protocol, design, and primary results of the clinical trial design can be found in our previous publication.^45^ Our current analysis included 70 eyes (34 metformin, 36 observation) from 44 participants (21 metformin, 23 observation) with GA area measurements at the baseline and ≥1 follow-up visit. One eye was excluded due to lack of baseline OCT imaging.

The study was conducted in accordance with the tenets of the Declaration of Helsinki and was approved by the institutional review board at each clinical site. The METforMIN trial obtained written informed consent at enrollment and complied with the Health Insurance Portability and Accountability Act.

### Outcome Measures and Baseline SFChT Grading

**Figure 1:**
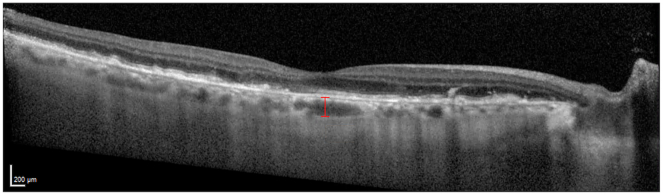
Measurement of subfoveal choroidal thickness on an OCT image for a representative eye. The vertical distance (marked in red) was manually measured at the fovea, extending from the hyperreflective line of Bruch membrane to the hyperreflective line of the choroidoscleral interface.

We performed a comprehensive eye examination during each visit and obtained OCT imaging. Two masked graders (A.V. and I.P.) independently measured SFChT using baseline OCT. We followed a previously described method by Lee et al.^12^ Using the caliper tool in the Heidelberg Eye Explorer Software (Heidelberg Engineering), we measured the vertical distance from the hyperreflective line of Bruch membrane to the hyperreflective line of the chorioscleral interface (Fig 1). The average of the 2 graders' measurements was used for statistical analysis. The 2 graders also performed a qualitative assessment of the presence of pachyvessels, defined as large choroidal vessel dilation with CC attenuation on baseline subfoveal OCT horizontal scans. In cases where the 2 graders disagreed, a third expert grader (L.L.S.) reviewed the scans and reached a final decision.^46^

**Figure 2:**
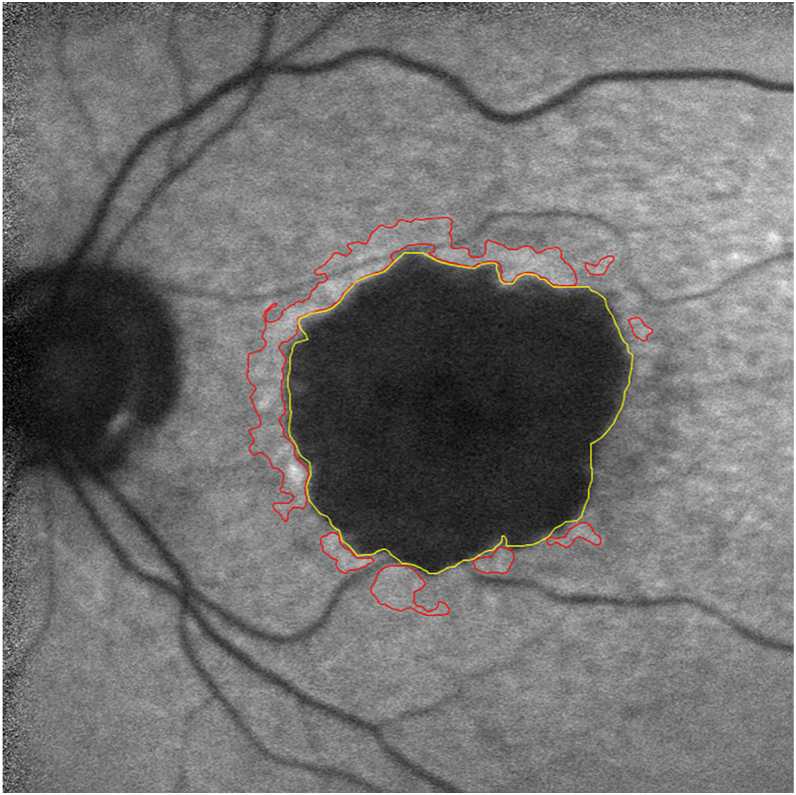
Blue autofluorescence image with superimposed delineation of GA in yellow and junctional hyperautofluorescent areas in red. Geographic atrophy grading was adopted from the METformin for the MINimization of Geographic Atrophy Progression trial and the shown hyperautofluorescent areas were delineated by the software. After correcting any perceived errors in the identified hyperautofluorescent areas, rim area focal hyperautofluorescence was calculated by dividing the total area in blue by the area interposed between a 450-mm border and the GA lesion. GA = geographic atrophy.

Rim area focal hyperautofluorescence signals were obtained from FAF images that were captured using a 488-nm excitation wavelength. Two masked graders (A.T.T. and A.Z.D) used a validated proprietary semiautomatic MATLAB software (The MathWorks, Inc)^47^ to identify the perilesional 450-μm border and potential hyperautofluorescent regions of interest (Fig 2). Detailed methods and results can be found in our previous publication.^34^ To summarize, the software drew a perimeter surrounding the GA lesions and applied a local threshold of +40 pixel value to the circumscribed region between the perimeter and the GA. Areas above this threshold were marked as hyperautofluorescent. Both graders then independently adjusted the segmentations in the software and then retraced hyperautofluorescent regions and GA lesions using ImageJ (National Institutes of Health)^48^ for analyses. Rim area focal hyperautofluorescence was calculated as the sum of all hyperautofluorescent areas within the 450-μm border divided by the total area enclosed between GA and its 450-μm border.^32^ For images with RAFH outside the 95% limits of agreement between the 2 graders, both graders assessed their gradings together, discussed their approach, and reached a mutual consensus. The intraclass correlation coefficient between the 2 graders after corrections was 0.78 (95% confidence interval, 0.72–0.83) with a mean difference of 0.016.

The primary outcome measure was perimeter-adjusted GA growth rate measured from FAF images. A prior study has shown that this metric reduces the confounding effects of morphological features such as lesion size, circularity, and lesion number, and can decrease the required sample size by > 50% compared with GA area growth rate.^44^ Using the Heidelberg Eye Explorer software, each FAF image was graded by 2 independent graders masked to treatment allocation. The graders measured the total area of GA lesions by manually tracing GA borders (Fig 2). We calculated GA area growth rate (mm^2^/year) by subtracting the GA area (mm^2^) at the first visit from the GA area (mm^2^) at the last visit and dividing the result by the time interval (years). We calculated the square root transformation of GA growth rate (mm/year) by subtracting the square root of GA at the first visit (mm) from the square root of GA at the last visit (mm) and dividing the result by the time interval (years). We calculated perimeter-adjusted GA area growth rate (mm/year) by dividing GA area growth rate (mm^2^/year) by the mean GA perimeter (mm) between the first and last visits.^44^ Our primary outcome was GA perimeter-adjusted growth rate, as we have previously demonstrated that adjusting the GA area growth rate with the GA perimeter accounts for the apparent effect of baseline GA area, number of lesions, and circularity index on GA enlargement rate.^44^

### Statistical Analysis

We used R software (version 4.1.1, R Foundation for Statistical Computing) to perform all statistical analyses. To assess intergrader reliability, we utilized a Bland–Altman plot and calculated the intraclass correlation coefficient. The mean SFChT between the 2 graders was used for all subsequent analyses. We performed Spearman rank correlation analysis to investigate the association between baseline SFChT and each of the following variables: baseline BCVA, baseline LLVA, baseline GA area, baseline RAFH signals, and perimeter-adjusted GA area growth rate. We then employed a linear mixed-effects regression model (“lme4” package in R software^49^) with a random intercept for participants to account for the correlation of eyes from the same individual. We performed univariable models investigating the association between 3 outcome measures and 9 baseline characteristics. The outcome measures included perimeter-adjusted GA growth rate, BCVA decline, and LLVA decline, and the baseline characteristics were age, sex, baseline SFChT, baseline GA area, baseline BCVA, baseline LLVA, baseline GA foveal involvement, baseline GA lesion configuration, and fellow eye GA status. Baseline GA foveal involvement was categorized as foveal involving (defined as GA involving the foveal center point) or foveal sparing (defined as GA not involving the foveal center point). Baseline GA lesion configuration was categorized as unifocal (defined as a single atrophic lesion in the eye) or multifocal (defined as ≥2 atrophic lesions in the eye). A multivariable linear mixed-effects model was then performed, investigating the association between the outcome measures and baseline SFChT, adjusting for age, sex, and baseline foveal involvement as covariates a priori, as well as other significant variables from the univariable models.

To further explore the effects of disease characteristics that may influence choroidal thickness relationships, we performed sensitivity analyses for the outcome measures of perimeter-adjusted GA growth rate, BCVA decline, and LLVA decline, stratified by GA phenotypes. Baseline GA size was stratified into 3 tertiles. Foveal involvement of GA was stratified into center-sparing versus foveal involvement. We also stratified lesion configuration into unifocal versus multifocal. For each stratified phenotype group, we modeled the outcome as a function of SFChT using a linear mixed-effects model. To investigate whether baseline SFChT mediates the treatment effect of oral metformin, we conducted an additional linear mixed-effects regression analysis of GA growth rate. This model included fixed effects for age, sex, treatment group, baseline SFChT, and an interaction term between treatment group and baseline SFChT. A random effect for participant was included to account for intereye correlations. The *P* values reported in our results were obtained from the respective linear mixed-effects models. For each outcome measure, we also performed a sensitivity analysis by including the pachyvessel classification in each linear mixed-effects model to assess if the presence of pachyvessels significantly changed our results.

## Results

### Participant Characteristics and Intergrader Reproducibility

**Figure 3:**
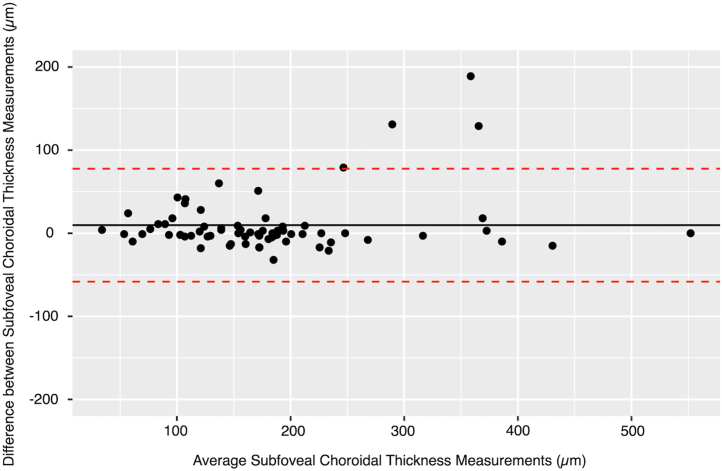
Bland–Altman plot comparing SFChT measurements between 2 graders (N = 70 images). The mean difference was 9.71 μm, with 95% limits of agreement ranging from –58.23 to 77.66 μm. The intraclass correlation coefficient for baseline SFChT was 0.930. SFChT = subfoveal choroidal thickness.

Seventy eyes (34 metformin; 36 observation) from 44 participants (21 metformin; 23 observation) were included in the final analysis. The time interval between the first and last visit was 6 months for 7 eyes, 12 months for 11 eyes, and 18 months for the remaining 52 eyes. The baseline characteristics of the participants are detailed in Table 1. The mean ± standard deviation SFChT at baseline was 186.8 ± 102.5 μm in the entire cohort, and 9 of 70 eyes (13%) had baseline SFChT ≥300 μm. At baseline, the SFChT was 178.9 ± 101.6 μm in 58 eyes with central GA, which was thinner but not statistically significantly different from the SFChT (225.1 ± 102.5 μm) in 12 eyes with noncentral GA (*P* = 0.85). The intraclass correlation coefficient between the 2 graders was 0.93 (95% confidence interval, 0.89–0.96) with a mean difference of 9.71 μm and Bland–Altman limits of agreement between –58.23 and 77.66 μm (Fig 3). Sixty-seven of 70 eyes (96%) had pachyvessels on baseline subfoveal horizontal OCT scans. Intergrader agreement was 97%.

**Table 1:** Baseline Characteristics of Participants in the Analysis

Baseline Characteristics of Participants in the Analysis	Observation	Metformin
Participants, n	23	21
Age, yrs, median (IQR)	78 (75–81)	74 (68–83)
Eyes, n	36	34
BCVA, letters, mean (SD)	60.3 (16.6)	61.0 (16.1)
LLVA, letters, mean (SD)∗	37.8 (14.9)	45.4 (16.7)
GA area, mm^2^, mean (SD)	8.1 (6.1)	5.8 (4.9)
Subfoveal choroidal thickness, μm, mean (SD)	201.9 (100.1)	172.6 (104.2)

BCVA = best-corrected visual acuity; GA = geographic atrophy; IQR = interquartile range; LLVA = low-luminance visual acuity; SD = standard deviation.

∗ Eight eyes in the observation group and 4 eyes in the metformin group did not have baseline LLVA.

### Association between Baseline SFChT and Baseline Outcomes

**Figure 4:**
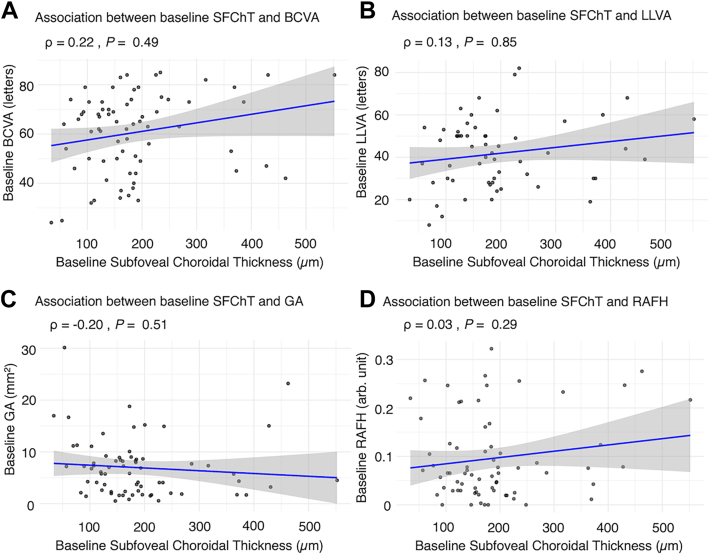
Spearman plots with correlation coefficients (ρ) and *P* values showing the association between baseline SFChT (μm) and **(A)** baseline GA area (mm^2^); **(B)** baseline BCVA (letters); **(C)** baseline LLVA (letters); and **(D)** baseline RAFH (arbitrary units). The blue line represents the trend line with the gray shaded area indicating the 95% CI. BCVA = best-corrected visual acuity; CI = confidence interval; GA = geographic atrophy; LLVA = low-luminance visual acuity; RAFH = rim area focal hyperautofluorescence; SFChT = subfoveal choroidal thickness.

Baseline SFChT was not significantly associated with baseline BCVA (letters; Spearman ρ = 0.22, *P* = 0.49). Similarly, no significant associations were observed between baseline SFChT and baseline LLVA (letters; Spearman ρ = 0.13, *P* = 0.85), GA area (mm^2^; Spearman ρ = –0.20, *P* = 0.51), or RAFH signals (arbitrary units; Spearman ρ = 0.03, *P* = 0.29) (Fig 4). The results for all baseline outcomes remain the same after including pachyvessel grading.

### Association between Baseline SFChT and GA Progression

**Figure 5:**
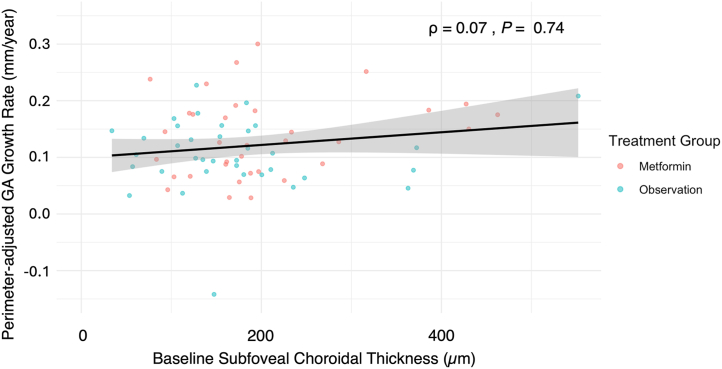
Plot illustrating the association between baseline subfoveal choroidal thickness (μm) and the perimeter-adjusted growth rate of GA (mm/year) (N = 70 eyes). No significant association was observed (ρ = 0.07; *P* = 0.74). The black line represents the trendline, with the gray shaded area indicating the 95% confidence interval. GA = geographic atrophy.

Univariable models investigating the association between our primary endpoint, GA perimeter-adjusted growth rate (mm/year) and the following baseline characteristics: age (years), sex, baseline SFChT (μm), baseline GA area (mm^2^), baseline BCVA (letters), baseline LLVA (letters), baseline GA foveal involvement, baseline GA lesion configuration, and fellow eye GA status, did not find a significant association between any of the baseline variables and perimeter-adjusted GA growth rate, aside from age (Table S1, available at www.ophthalmologyscience.org). After adjusting for age and sex, perimeter-adjusted GA growth rate was not significantly associated with baseline SFChT (μm; Spearman ρ = 0.07, *P =* 0.74) (Fig 5). Similarly, baseline SFChT was not significantly associated with the growth rate of GA area (mm^2^/year; Spearman ρ = –0.007, *P* = 0.74), or the square root–transformed GA area growth rate (mm/year; Spearman ρ = 0.07, *P =* 0.76). Univariable analysis showed no significant associations between baseline characteristics and BCVA decline (Table S2, available at www.ophthalmologyscience.org). Univariable analysis of the association between baseline characteristics and LLVA decline only showed baseline LLVA as a significant independent predictor for LLVA decline (Table S3, available at www.ophthalmologyscience.org). After adjusting for age and sex, baseline SFChT was not associated with the decline rate of BCVA *(P* = 0.14) or the decline rate of LLVA (*P* = 0.71). The results for all growth rate measurements remain the same after including pachyvessel grading. Sensitivity analyses exploring the effects of GA disease characteristics on choroidal thickness relationships found that in eyes with GA involving the fovea, thicker SFChT (μm) was significantly associated with less severe BCVA decline (letters; Spearman ρ = 0.03, *P* = 0.03) (Table 2). Baseline GA area size, baseline GA foveal involvement, and baseline GA lesion configuration did not significantly mediate the relationship between SFChT and GA growth rate and LLVA decline (Tables S4 and S5, available at www.ophthalmologyscience.org). Baseline GA area size and baseline GA lesion configuration did not significantly mediate the relationship between SFChT and BCVA decline (Table 2).

**Table 2:** Association between Baseline Subfoveal Choroidal Thickness and Best-Corrected Visual Acuity Decline (Letters) in Geographic Atrophy Subgroups

Subgroup	N	Spearman ρ	Estimate	95% CI	*P* Value
GA size					
Small	24	–0.13	0.011	–0.015, 0.038	0.37
Medium	23	0.09	0.009	–0.016, 0.035	0.47
Large	23	0.04	0.008	–0.019, 0.035	0.53
Foveal involvement					
Foveal involving	58	0.03	0.019	0.002, 0.036	0.03
Foveal sparing	12	–0.19	–0.013	–0.048, 0.021	0.39
GA lesion configuration					
Multifocal	46	0.03	0.013	–0.002, 0.029	0.09
Unifocal	24	–0.13	0.003	–0.027, 0.034	0.81

CI = confidence interval; GA = geographic atrophy.

### Influence of Baseline SFChT on the Treatment Effect of Oral Metformin

We did not find a significant difference in the GA perimeter-adjusted growth rate between the oral metformin group and observation group after adjusting for baseline SFChT (μm; *P* = 0.43). In addition, baseline SFChT (μm) did not significantly influence the effect of oral metformin on GA perimeter-adjusted growth rate (mm/year; *P =* 0.78). The result did not change after including pachyvessel grading.

## Discussion

Despite the growing interest in imaging biomarkers for GA, the role of SFChT remains poorly understood. Prior studies have reported conflicting results regarding the association between SFChT and GA severity, visual function, and disease progression.^9^, ^10^, ^11^, ^12^^,^^21^, ^22^, ^23^, ^24^, ^25^, ^26^^,^^35^^,^^50^^,^^51^ Our study addresses this gap by performing a comprehensive analysis of the relationship between SFChT and variable anatomical and functional outcomes in GA.

We found no significant association between baseline SFChT and baseline outcomes, including BCVA, LLVA, GA area, foveal involvement, or RAFH signal. Furthermore, SFChT was not significantly associated with GA growth rate—whether measured as unadjusted, square root–transformed, or perimeter-adjusted. Nor did SFChT mediate the effect of oral metformin on GA progression. These findings suggest that SFChT is not a reliable biomarker for GA progression across a broad patient population. However, a key finding of our study was that greater baseline SFChT was significantly associated with slower BCVA decline in eyes with foveal-involving GA, indicating a potential localized protective role of choroidal thickness in preserving central vision.

The correlation between SFChT and slower BCVA decline in foveal-involving GA is consistent with earlier reports that link thicker choroid to better BCVA in GA.^12^^,^^21^ These results suggest that SFChT may play a localized role in preserving central visual function. One possible explanation is that choroidal thinning reflects reduced blood flow and metabolic support to the macula, contributing to BCVA loss.^12^ This localized role is also supported by OCT angiography studies showing that CC flow deficits near GA margins are linked to faster GA growth.^52^^,^^53^, ^54^, ^55^, ^56^, ^57^ Although we did not find a significant link between SFChT and perimeter-adjusted GA growth rate in foveal-involving GA, this may reflect the localized nature of SFChT's protective effect, which would not necessarily predict progression beyond the foveal region. We also found no significant association between SFChT and LLVA decline, even after sensitivity analysis for foveal involvement. This could be due in part to limited LLVA data, reducing statistical power. More importantly, SFChT would be expected to have the strongest relevance to BCVA, which reflects central retinal function.^58^, ^59^, ^60^, ^61^ In contrast, LLVA depends more on rod photoreceptor function, which is concentrated in the parafoveal and peripheral retina.^62^, ^63^, ^64^

Current literature suggests that choroidal perfusion is important to the health of retinal pigment epithelium cells.^65^, ^66^, ^67^, ^68^, ^69^, ^70^, ^71^ Changes in choroidal blood supply may play a role in the pathogenesis of AMD and other ocular diseases, leading to significant interest in identifying biomarkers to quantify choroidal vasculature.^8^^,^^13^^,^^65^^,^^66^^,^^52^, ^72^, ^73^ A reliable biomarker for choroidal vascular health can lead to a better understanding of disease pathogenesis and inform early diagnosis and treatment of AMD. Few studies have looked at the relationship between SFChT and GA progression. Lee et al^12^ found a negative correlation between SFChT and GA area growth rate; however, their study did not adjust for GA lesion size or configurations using square root transformation or perimeter-adjusted growth rates. These adjusted measures of GA growth rate are more robust in accounting for the influence of baseline GA lesion size on GA progression.^4^^,^^38^^,^^44^^,^^74^, ^75^, ^76^, ^77^ Other studies employing square root transformation as an outcome measure found no significant association between SFChT and GA progression.^13^^,^^35^ In our study, we analyzed perimeter-adjusted growth rate, square root transformation, and unadjusted GA growth rate to comprehensively assess the association between SFChT and GA progression. With a larger sample size, our findings indicate that SFChT does not significantly influence GA progression when using adjusted growth rate measures. When adding sensitivity analyses for different phenotypes of GA, including foveal involvement and lesion configuration, SFChT still lacked a significant association with GA progression, suggesting that SFChT may have limited prognostic value for overall GA progression.

Taken together, these findings suggest that while SFChT may have prognostic value for central visual outcomes, it is not a robust biomarker for GA progression overall. This helps explain inconsistencies in the literature. Subfoveal choroidal thickness may not accurately reflect choroidal vascularity, because it does not distinguish vascular from stromal components—vascular loss could be masked by stromal thickening.^72^^,^^73^ OCT angiography may provide more robust markers for localized choroidal vasculature, including information about CC flow deficits and vessel densities surrounding GA margins,^13^^,^^65^^,^^52^ which were unavailable in our cohort, limiting our ability to investigate the association between the CC and GA progression. Future studies examining other vascular-specific metrics, such as choroidal vascular index, may provide a more accurate understanding of the vascular contributions to GA progression. Additionally, future studies examining localized changes to choroidal thickness at GA margins may better elucidate the localized protective role of choroidal thickness and its impact on GA enlargement.

Our study is not without other limitations. First, several studies have found that axial length is inversely associated with SFChT in both highly myopic and healthy eyes.^78^, ^79^, ^80^, ^81^, ^82^, ^83^ We did not measure axial length at the time of the patients' visits and were thus unable to adjust for axial length as a covariate in our analysis. Subfoveal choroidal thickness has also been shown to be influenced by several baseline factors such as systolic blood pressure, ocular perfusion pressure, and ischemic heart disease, which we did not collect in our initial trial. This may potentially obscure the relationship between SFChT and GA growth rate. Second, although our sample size of 70 eyes is larger than that in most prior studies investigating the relationship between SFChT and GA progression, it remains relatively small, limiting the statistical power of our study. Third, while 53 of 70 eyes (74.3%) had 18 months of follow-up, 25.7% of participants dropped from the study or missed interval visits and thus had shorter follow-up intervals. The retention rate of the METforMIN trial is comparable to that of previous phase III trials; 74% and 76% of patients remained in the OAKS and DERBY trials, 2 large global phase III trials, respectively, at 24 months.^84^ Our study also did not look at regional changes in choroidal thickness and focused on SFChT as a potential biomarker for GA progression, as several previous studies suggested that SFChT is a potential biomarker for GA severity and progression rate.^10^^,^^12^^,^^26^^,^^50^ Our findings suggest that SFChT may not be of robust prognostic value for overall GA progression but may have a more important localized role. We propose future studies to investigate choroidal changes at or around GA margins, which may improve our understanding of the utility of choroidal thickness as a biomarker for GA progression.

Additionally, other studies have identified a relationship between choroidal thickness and GA in patients with pachychoroid, generally defined as SFChT > 300 μm.^85^ Few eyes in our study had baseline SFChT > 300 μm, which precluded us from investigating whether patients with pachychoroid are associated with lower GA progression. Our study also included a sensitivity analysis to assess whether the presence of pachyvessels significantly influenced our results. However, this analysis was limited by the fact that 96% of eyes in our cohort showed pachyvessels on the baseline subfoveal OCT horizontal scan. While Baek et al^46^ reported the presence of pachyvessels in patients with neovascular AMD and all stages of nonneovascular AMD, no studies to date have reported the prevalence of pachyvessels specifically in eyes with GA to the best of our knowledge. Further research is needed to investigate the association between pachyvessels and GA.

In conclusion, greater baseline SFChT was significantly associated with a slower rate of BCVA decline in eyes with foveal-involving GA, suggesting a possible localized role of choroidal thickness in preserving central vision. In contrast, baseline SFChT was not significantly associated with GA growth rate—whether perimeter-adjusted, unadjusted, or square root–transformed—or with LLVA decline, nor with baseline anatomical or functional measures, including GA area and hyperautofluorescence signal. Baseline SFChT also did not significantly mediate the effect of oral metformin on GA progression. Overall, while SFChT lacks prognostic value for GA progression in the broader patient population, it may hold limited relevance for central visual outcomes in foveal-involving disease and warrants cautious consideration in clinical trial design.
