# Supplementary material for: Association between Baseline Subfoveal Choroidal Thickness and Anatomical and Functional Outcomes in Geographic Atrophy
Source: Ophthalmol Sci. 2025 Oct 27;6(2):100986. doi: 10.1016/j.xops.2025.100986 (PMC12720346; doi:10.1016/j.xops.2025.100986)
Supplement: Table S1 [file mmc1.pdf]

Supplementary Table 1. Univariable Analysis for Perimeter-adjusted Geographic Atrophy Growth Rate (mm/year)

Variable	N	Estimate	95% CI*	p-value
Sex				
Female	22	—	—	—
Male	48	0.01	-0.03, 0.06	0.52
Age (years)	70	-0.003	-0.005, -0.0004	0.03
Baseline Subfoveal Choroidal Thickness (μm)	70	0.0001	-0.0001, 0.0003	0.41
Baseline GA† Area (mm^2)	70	-0.001	-0.004, 0.002	0.45
Baseline BCVA‡ (letters)	70	0.0003	-0.0007, 0.0012	0.56
Baseline LLVA§ (letters)	58	0.0005	-0.0004, 0.0015	0.28
Baseline Foveal Involvement				
Foveal Sparing	12	—	—	—
Foveal Involving	58	-0.02	-0.07, 0.02	0.27
Baseline Lesion Configuration				
Unifocal	24	—	—	—
Multifocal	46	-0.02	-0.05, 0.01	0.27
Fellow Eye GA† Status				
No	1	—	—	—
Yes	69	0.08	-0.06, 0.23	0.27

*CI = Confidence Interval
†GA = Geographic Atrophy
‡BCVA = Best-corrected Visual Acuity
§LLVA = Low luminance visual acuity
