# Supplementary material for: Association between Baseline Subfoveal Choroidal Thickness and Anatomical and Functional Outcomes in Geographic Atrophy
Source: Ophthalmol Sci. 2025 Oct 27;6(2):100986. doi: 10.1016/j.xops.2025.100986 (PMC12720346; doi:10.1016/j.xops.2025.100986)
Supplement: Table S2 [file mmc2.pdf]

Supplementary Table 2. Univariable Analysis for Best-corrected Visual Acuity Decline (letters)

Variable	N	Estimate	95% CI*	p-value
Sex				
Female	22	—	—	—
Male	48	-1.40	-4.70, 2.00	0.41
Age (years)	70	-0.07	-0.23, 0.09	0.41
Baseline Subfoveal Choroidal Thickness (μm)	70	0.01	-0.01, 0.02	0.14
Baseline GA† Area (mm^2)	70	-0.04	-0.34, 0.25	0.78
Baseline BCVA‡ (letters)	70	-0.02	-0.13, 0.10	0.79
Baseline LLVA§ (letters)	58	0.06	-0.03, 0.15	0.17
Baseline Foveal Involvement				
Foveal Sparing	12	—	—	—
Foveal Involving	58	-2.46	-6.36, 1.43	0.22
Baseline Lesion Configuration				
Unifocal	18	—	—	—
Multifocal	40	1.28	-1.69, 4.23	0.40
Fellow Eye GA† Status				
No	1	—	—	—
Yes	69	-8.1	27.0, 11.0	0.41

*CI = Confidence Interval
†GA = Geographic Atrophy
‡BCVA = Best-corrected Visual Acuity
§LLVA = Low luminance visual acuity
