# Supplementary material for: Association between Baseline Subfoveal Choroidal Thickness and Anatomical and Functional Outcomes in Geographic Atrophy
Source: Ophthalmol Sci. 2025 Oct 27;6(2):100986. doi: 10.1016/j.xops.2025.100986 (PMC12720346; doi:10.1016/j.xops.2025.100986)
Supplement: Table S3 [file mmc3.pdf]

Supplementary Table 3. Univariable Analysis for Low Luminance Visual Acuity Decline (letters)

Variable	N	Estimate	95% CI[*]	p-value
Sex				
Female	19	—	—	—
Male	39	1.10	-3.60, 5.80	0.65
Age (years)	58	-0.15	-0.39, 0.09	0.21
Baseline Subfoveal Choroidal Thickness (μm)	58	0.003	-0.016, 0.023	0.73
Baseline GA[†] Area (mm^2)	58	-0.27	-0.69, 0.14	0.19
Baseline BCVA[‡] (letters)	58	-0.07	-0.21, 0.07	0.32
Baseline LLVA[§] (letters)	58	-0.19	-0.34, 0.04	0.02
Baseline Foveal Involvement				
Foveal Sparing	12	—	—	—
Foveal Involving	46	3.48	-1.92, 8.81	0.21
Baseline Lesion Configuration				
Unifocal	18	—	—	—
Multifocal	40	0.87	-3.44, 5.19	0.39
Fellow Eye GA[†] Status				
No	1	—	—	—
Yes	57	-7.3	-31.0, 16.0	0.54

[*]CI = Confidence Interval
[†]GA = Geographic Atrophy
[‡]BCVA = Best-corrected Visual Acuity
[§]LLVA = Low luminance visual acuity
