# Supplementary material for: Association between Baseline Subfoveal Choroidal Thickness and Anatomical and Functional Outcomes in Geographic Atrophy
Source: Ophthalmol Sci. 2025 Oct 27;6(2):100986. doi: 10.1016/j.xops.2025.100986 (PMC12720346; doi:10.1016/j.xops.2025.100986)
Supplement: Table S4 [file mmc4.pdf]

Supplementary Table 4. Association Between Baseline Subfoveal Choroidal Thickness and Perimeter-adjusted Geographic Atrophy Growth Rate (mm/year) in Geographic Atrophy Subgroups

**Subgroup**	**N**	**Spearman's ρ**	**Estimate**	**95% CI***	**p-value**
GA† Size					
Small	24	-0.07	-0.0001	-0.0006, 0.0002	0.42
Medium	23	0.30	0.0001	-0.0002, 0.0003	0.66
Large	23	-0.01	0.0001	-0.0003, 0.0006	0.65
Foveal Involvement					
Foveal Involving	58	0.02	0.0001	-0.0002, 0.0003	0.42
Foveal Sparing	12	0.16	-0.0002	-0.0007, 0.0002	0.37
GA† Lesion Configuration					
Multifocal	46	-0.02	-0.0001	-0.0003, 0.0001	0.58
Unifocal	24	0.25	0.0002	-0.0002, 0.0006	0.31

*CI = Confidence Interval
†GA = Geographic Atrophy
