# Supplementary material for: Association between Baseline Subfoveal Choroidal Thickness and Anatomical and Functional Outcomes in Geographic Atrophy
Source: Ophthalmol Sci. 2025 Oct 27;6(2):100986. doi: 10.1016/j.xops.2025.100986 (PMC12720346; doi:10.1016/j.xops.2025.100986)
Supplement: Table S5 [file mmc5.pdf]

Supplementary Table 5. Association Between Baseline Subfoveal Choroidal Thickness and Low Luminance Visual Acuity Decline (letters) in Geographic Atrophy Subgroups

Subgroup	N	Spearman's ρ	Estimate	95% CI*	p-value
GA† Size					
Small	21	-0.13	-0.002	-0.040, 0.037	0.92
Medium	22	0.20	-0.002	-0.038, 0.034	0.91
Large	16	0.21	0.013	-0.019, 0.044	0.38
Foveal Involvement					
Foveal Involving	46	0.06	0.021	-0.006, 0.048	0.12
Foveal Sparing	12	-0.03	-0.015	-0.047, 0.017	0.33
GA† Lesion Configuration					
Multifocal	40	0.08	-0.001	-0.028, 0.026	0.95
Unifocal	18	0.16	0.010	-0.021, 0.041	0.48

*CI = Confidence Interval
†GA = Geographic Atrophy
